# Supplementary material for: Interplay between Podoplanin, CD44s and CD44v in Squamous Carcinoma Cells
Source: Cells. 2020 Sep 29;9(10):2200. doi: 10.3390/cells9102200 (PMC7601683; doi:10.3390/cells9102200)
Supplement: Supplementary file 1 [file cells-09-02200-s001.pdf]

Supplementary Material

Table S1. Description of Mouse Skin Carcinogenesis Cell Lines.

Cell Line	Origin	Mutations	Phenotype	Culture Medium	Reference
**MCA3D**	Primary keratinocytes treated with DMBA	-	E	Hams'F12	[1]
**PB**	DMBA/TPA induced papilloma	-	E	Hams'F12	[2]
**PDV**	Primary keratinocytes transformed with DMBA *in vitro*	H-ras; p53	E	Hams'F12	[3,4]
**MSC11 B9**	DMBA-induced carcinoma	H-ras; p53	E	DMEM	[5]
**MSC11 A5**	DMBA-induced carcinoma	H-ras; p53	F	DMEM	[5]
**CarcC**	DMBA/TPA-induced cacinoma	H-ras	F	DMEM	[6]
**CarB**	DMBA/TPA-induced cacinoma	H-ras	F	DMEM	[6]

E, epitelial; F, fibroblastic

Table S2. Description of Human SCC Cell Lines

Cell Line	Origin	Culture medium
**HaCaT**	Normal Immortalized keratinocytes	DMEM
**HN30**	Pharyngeal squamous cell carcinoma	DMEM
**HN19**	Head and neck squamous cell carcinoma derived from metastatic site: Lymph node	DMEM
**HN5**	Squamous cell carcinoma of the oral tongue	DMEM
**A253**	Epidermoid carcinoma from the submaxillary salivary gland	DMEM
**Fadu**	Pharyngeal squamous cell carcinoma	DMEM
**SCC13**	Skin squamous cell carcinoma	Keratinocyte-SFM
**HEK293T**	Human embryonic kidney cells	DMEM

Table S3.- cDNA Constructs for CD44 isoforms

Constructs	Template	Oligonucleotides	Restriction sites	Final Plasmids
**CD44sHa**	HT1080	5′ CCGTTCGCTCGAATTCCCATGGACAAGTTT 3′<br>5′	EcoRI XhoI	pcDNA3-Ha

		AAGATAATGGTGTAGCTCGAGCACCCC AATCTTCAT 3′		
**CD44v3-10-Ha**	A253	5′ CCGTTCGCTCGAATTCCCATGGACAAGT TT3′<br>5′ AAGATAATGGTGTAGCTCGAGCACCCC AATCTTCAT 3′	EcoRI XhoI	pcDNA3 -Ha
**CD44v6-10-Ha**	HN5	5′ CCGTTCGCTCGAATTCCCATGGACAAGT TT 3′<br>5′ AAGATAATGGTGTAGCTCGAGCACCCC AATCTTCAT 3′	EcoRI XhoI	pcDNA3 -Ha
**CD44v8-10-Ha**	HN5	5′ CCGTTCGCTCGAATTCCCATGGACAAGT TT 3′<br>5′ AAGATAATGGTGTAGCTCGAGCACCCC AATCTTCAT 3′	EcoRI XhoI	pcDNA3 -Ha
**CD44sC9-Ha**	HN5	5′CCGTTCGCTCGAATTCCCATGGACAAG TTT3′<br>5′ACCTGAATCCTCGAGACTTCTTCGACT GTTGAC3′	EcoRI xhoI	pcDNA3 -Ha
**CD44s-eGFP**	HT1080	5′CCGTTCGCTCGAATTCCCATGGACAAG TTT3′<br>5′ATAATGGTGTAGGGGTACCACCCCAA TCTTCAT3′	EcoRI KpnI	pEGFP- N1
**CD44v3-10-eGFP**	A253	5′CCGTTCGCTCGAATTCCCATGGACAAG TTT3′<br>5′ATAATGGTGTAGGGGTACCACCCCAA TCTTCAT3′	EcoRI KpnI	pEGFP- N1
**CD44v6-10-eGFP**	HN5	5′CTCAAGCTTCGAATTCATGGACAAGTT TTGGTGGCAC3′<br>5′GATCCCGGGCCCGCGGTACCCCCACC CCAATCTTCATGTCCAC3′	EcoRI KpnI	pEGFP- N1
**CD44v8-10-eGFP**	HN5	5′CCGTTCGCTCGAATTCCCATGGACAAG TTT3′<br>5′ATAATGGTGTAGGGGTACCACCCCAA TCTTCAT3′	EcoRI KpnI	pEGFP- N1
**CD44sC9-eGFP**	HN5	5′CCGTTCGCTCGAATTCCCATGGACAAG TTT3′<br>5′ACCTGAAGGTACCCAACTTCTTCGACT GTTGAC3′	EcoRI KpnI	pEGFP- N1

Table S4.- Wild Type and Mutant Podoplanin cDNA Constructs

Constructs	Template	Oligonucleotides	Restriction sites	Final Plasmids
**PDPN-mCherry**	PDPN-eGFP	-	EcoRI KpnI	pmCherry-N1
**PDPN-Flag**	pcDNA3-PDPN [7]	5′ AGCTTCGAATTCCCCGATGTGG 3′<br>5′ TTGCTCACCTCGAGGGCGAC 3′	EcoRI XhoI	pcDNA3-Flag
**PDPN-ΔEC-Flag**	PDPN-ΔEC-eGFP [8]	5′ CGTCAGATCGGTACCCATGTGG 3′<br>5 ′TTGAGCGAATTCTCTGAGTCCGGA3′<br>5′ ATGGTTTGGAATTCGTGACCCTGGTT 3′<br>5′ TTCAGCCTCGAGGGGCGAGTACCT 3′	KpnI EcoRI EcoRI XhoI	pcDNA3-Flag
**PDPN-ΔEC QN.N-Flag**	pcDNA3-PDPN ΔEC-Flag	QN<br>5′ GTTATGCAAAACATGTCGGGAAGGTAC 3′<br>5′ GTTTTGACATGCATAACCACAACGATG 3′<br>N<br>5′ GGAAACTACTCGCCCCTCG 3′<br>5′ GAGTAGTTTCCCGACATGTTTTG 3′	-	pcDNA3-Flag
**PDPN-ΔPLAG3-Flag**	pcDNA3-PDPNFlag	5′ GGTGCCACCAGCGAAGACCGCTATAAG 3′<br>5′ CGCTGGTGGCACCTGGCATGGC 3′	-	pcDNA3-Flag
**PDPN-PLAG3m-Flag**	pcDNA3-PDPNFlag	5′ GTGCCGCAGCTGATGTGGTGGCTCCAGGAACCAGC 3′<br>5′ CTGGAGCCACCACATCAGCTGCGGCACCTGGCATGG 3′	-	pcDNA3-Flag
**PDPN-S/Tm-Flag**	pcDNA3-PDPNFlag	5′ AGCTTCGAATTCCCCGATGTGG 3′<br>5′ TTGCTCACCTCGAGGGCGAC 3′	EcoRI XhoI	pcDNA3-Flag
**PDPN-PLAG3Tm-Flag**	pcDNA3-PDPN-Flag	5′ GGTGGCTCCAGGAACCAG 3′<br>5′ GGAGCCACCACATCATC 3′	-	pcDNA3-Flag
**PDPN-PLAG4Tm-Flag**	pcDNA3-PDPN-Flag	5′ GCCAGCTTCAGAAAGCAC 3′<br>5′ GAAGCTGGCAGATCCTC 3′	-	pcDNA3-Flag
**PDPN-I1-Flag**	pcDNA3-PDPN-Flag	T65-T66<br>5′ CTTGGCAGCTCTGGTGGCAAC 3′<br>5′ CAGAGCTGCCAAGCCAGACTTATAG 3′<br>T70-T76<br>5′ GGCAGCAGCTGTCAACGCTGTAGCAGG	-	pcDNA3-Flag

		CATTCGCA TC3′ 5′ CCTGCTACAGCGTTGACAGCTGCTGCCA CCAGAGC 3′		
**PDPN-I2-Flag**	pcDNA3-PDPN-Flag	S98-T100 5′ GTCCAGCCGCCGCAGCCTCAAACG 3′ 5′ GCTGCGGCGGCTGGACTTTGTTCTTG 3′ S107-T110 5′ CACCGCTCACGCCGCGGAGAAAGTGGA TG 3′ 5′ CTCCGCGGCGTGAGCGGTGGCCACGTTT G 3′	-	pcDNA3-Flag
**PDPN-TMCD45-Flag**	PDPN-TMCD45-eGFP [9]	5′ AGCTTCGAATTCCCCGATGTGG 3′ 5′ TTGCTCACCTCGAGGGCGAC 3′	EcoRI XhoI	pcDNA3-Flag
**PDPN-TMSYN-Flag**	pcDNA3-PDPN-Flag	5′TACTACGTCGACAATGATGATCATCTT GGGAGTGATTTGC3′ 5′TACTACTCCGGACATTTTTCGGAAGTA AACTATGATGATGATGAGG3′	-	pcDNA3-Flag
**PDPN-TMGPA-Flag**	pcDNA3-PDPN-Flag	5′ TACTACGTCGACAATAACACTCATTATT TTTGG 3′ 5′ TACTACTCCGGACATTTTTCGAATACCG TAAGAAATT AAGAGG 3′	-	pcDNA3-Flag
**PDPN-G137L-Flag**	PDPN-G137L-eGFP [9]	5′ AGCTTCGAATTCCCCGATGTGG 3′ 5′ TTGCTCACCTCGAGGGCGAC 3′	EcoRI XhoI	pcDNA3-Flag
**PDPN-ΔCT-Flag**	PDPN-ΔCTeGFP [8]	5′ AGCTTCGAATTCCCCGATGTGG 3′ 5′ TTGCTCACCTCGAGGGCGAC 3′	EcoRI XhoI	pcDNA3-Flag
**PDPN-QN.N-Flag**	PDPN-QNN-eGFP [8]	5′ AGCTTCGAATTCCCCGATGTGG 3′ 5′ TTGCTCACCTCGAGGGCGAC 3′	EcoRI XhoI	pcDNA3-Flag

Table S5.- Oligonucleotides Used for RT-PCR

Name	Oligonucleotides
**hs3**	5′ TTTGCTCCACCTTCTTGACTCC 3′
**hs5**	5′ GATGGAGAAAGCTCTGAGCATC 3′
**C5**	5′ AAGACATCTACCCCAGCAAC 3′
**v2**	5′ GATGAGCACTAGTGCTACAG 3′
**v3a**	5′ ACGTCTTCAAATACCATCTC 3′
**v3b**	5′ TGGGAGCCAAATGAAGAAAA 3′
**v4**	5′ TCAACCACACCACGGGCTTT 3′
**v5**	5′ GTAGACAGAAATGGCACCAC 3′
**v6**	5′ CAGGCAACTCCTAGTAGTAC 3′
**v7**	5′ CAGCCTCAGCTCATACCAGC 3′
**v8**	5′ TCCAGTCATAGTATAACGCT 3′

**v9**	5′ CAGAGCTTCTCTACATCACA 3′
**v10**	5′ GGTGGAAGAAGAGACCCAAA 3′
**C9**	5′ ACCTGAAGGTACCCAACTTCTTCGACTGTTGAC 3′
**ms5**	5′ CAACCGTGATGGTACTCGCT 3′
**ms3**	5′ ATGAGTCACAGTGCGGGAAC 3′
**Human PDPN Fw**	5′ CGGGAACGATGTGGAAGGTGTCA 3′
**Human PDPN Rv**	5′ GGGACAGGGCACAGAGTCAGAAAC 3′
**Mouse PDPN Fw**	5′ AAAAACCCACTAGCTGCTGAGGCTCCAA 3′
**Mouse PDPN Rv**	5′ ATGGGTCATCTTCCTCCACAGGAAGAGG 3′
**β-actin Fw**	5′ GTGGGCCGCTCTAGGCACCAA 3′
**β-actin Rv**	5′ CTCTTTGATGTCACGCAGGATTTC 3′
**GAPDH Fw**	5′ TGAAGGTCGGTGTGAACGGATTTGGC 3′
**GAPDH Rv**	5′ CATGTAGGCCATGAGGTCCACCAC 3′

REFERENCES

1. Kulesz-Martin, M.; Kilkenny, A.E.; Holbrook, K.A.; Digernes, V.; Yuspa, S.H. Properties of carcinogen altered mouse epidermal cells resistant to calcium-induced terminal differentiation. *Carcinogenesis* **1983**, *4*, 1367–1377, doi:10.1093/carcin/4.11.1367.
2. Yuspa, S.H.; Morgan, D.; Lichti, U.; Spangler, E.F.; Michael, D.; Kilkenny, A.; Hennings, H. Cultivation and characterization of cells derived from mouse skin papillomas induced by an initiation-promotion protocol. *Carcinogenesis* **1986**, *7*, 949–958, doi:10.1093/carcin/7.6.949.
3. Pons, M.; Cigudosa, J.C.; Rodríguez-Perales, S.; Bella, J.L.; González, C.; Gamallo, C.; Quintanilla, M. Chromosomal instability and phenotypic plasticity during the squamous–spindle carcinoma transition: association of a specific T(14;15) with malignant progression. *Oncogene* **2005**, *24*, 7608–7618, doi:10.1038/sj.onc.1208903.
4. Quintanilla, M.; Haddow, S.; Jones, D.; Jaffe, D.; Bowden, G.T.; Balmain, A. Comparison of ras activation during epidermal carcinogenesis in vitro and in vivo. *Carcinogenesis* **1991**, *12*, 1875–1881, doi:10.1093/carcin/12.10.1875.
5. Burns, P.A.; Kemp, C.J.; Gannon, J.V.; Lane, D.P.; Bremner, R.; Balmain, A. Loss of heterozygosity and mutational alterations of the p53 gene in skin tumours of interspecific hybrid mice. *Oncogene* **1991**, *6*, 2363–2369.
6. Buchmann, A.; Ruggeri, B.; Klein-Szanto, A.J.P.; Balmain, A. Progression of Squamous Carcinoma Cells to Spindle Carcinomas of Mouse Skin Is Associated with an Imbalance of H-ras Alleles on Chromosome 7. *Cancer Res.* **1991**, *51*, 4097–4101.
7. Martín-Villar, E.; Scholl, F.G.; Gamallo, C.; Yurrita, M.M.; Muñoz-Guerra, M.; Cruces, J.; Quintanilla, M. Characterization of human PA2.26 antigen (T1alpha-2, podoplanin), a small membrane mucin induced in oral squamous cell carcinomas. *Int. J. Cancer* **2005**, *113*, 899–910, doi:10.1002/ijc.20656.
8. Martín-Villar, E.; Megías, D.; Castel, S.; Yurrita, M.M.; Vilaró, S.; Quintanilla, M. Podoplanin binds ERM proteins to activate RhoA and promote epithelial-mesenchymal transition. *J. Cell Sci.* **2006**, *119*, 4541–4553, doi:10.1242/jcs.03218.

9. Fernández-Muñoz, B.; Yurrita, M.M.; Martín-Villar, E.; Carrasco-Ramírez, P.; Megías, D.; Renart, J.; Quintanilla, M. The transmembrane domain of podoplanin is required for its association with lipid rafts and the induction of epithelial-mesenchymal transition. *Int. J. Biochem. Cell Biol.* **2011**, *43*, 886–896, doi:10.1016/j.biocel.2011.02.010.
